# Herpes Simplex Virus Necrotic Lymphadenitis Masquerading as Lymphoma

**DOI:** 10.7759/cureus.62485

**Published:** 2024-06-16

**Authors:** Ismail O Malik, Meenal Malviya

**Affiliations:** 1 Infectious Diseases, Ascension St. John Hospital, Grosse Pointe Woods, USA

**Keywords:** lymphadenitis, diffuse large b-cell lymphoma, chronic lymphocytic leukemia, lymphadenopathy, herpes simplex virus

## Abstract

In immunocompromised patients, the rapid development of lymphadenopathy could pose a few diagnostic challenges. This is important, especially with chronic lymphocytic leukemia and small lymphocytic lymphoma (CLL/SLL) which can manifest with the development of infections or may even progress with transformation into a more aggressive form of the disease. We report a case of a patient with CLL/SLL who presented with fever and worsening dyspnea as well as inguinal lymphadenopathy upon evaluation. The excisional biopsy of affected lymph nodes revealed herpes simplex virus lymphadenitis confirmed by immunohistochemical staining. Flow cytometry showed no progression to diffuse large B-cell lymphoma. This case highlights the importance of considering a broad spectrum of differential diagnoses when assessing lymphadenopathy in immunocompromised patients receiving active immunosuppressive therapy.

## Introduction

The pathogenesis of opportunistic infections (bacterial, fungal, and viral) in CLL/SLL patients is wide-ranging. Infections can be secondary to the effects of hypogammaglobulinemia, cell-mediated immunity, complement deficiencies, neutrophilic and phagocytic defects, and from the adverse effects of immunosuppressive drugs such as alkylating agents, purine analogs, tyrosine kinase inhibitors (TKIs), corticosteroids, and monoclonal antibodies [1].

Herpes simplex viruses (HSV) 1 and 2 represent two of the eight human pathogenic herpes viruses known, along with varicella-zoster virus (VZV), cytomegalovirus (CMV), Epstein-Barr virus (EBV), human herpesvirus 6 (variants A and B), human herpesvirus 7 and Kaposi sarcoma virus or human herpesvirus 8 [2]. They usually establish latency in sensory neurons or lymphocytes and later reactivate with varying degrees of organ-system involvement [2]. 

In CLL/SLL patients, a rare but aggressive histologic transformation known as Richter transformation (RT) can occur. This situation can eventually lead to either a diffuse large B cell lymphoma (DLBCL) or, rarely, a classic Hodgkin lymphoma (CHL) variant. On the other hand, HSV reactivation can also occur in immunocompromised patients, although rarely associated with rapid lymph node development mimicking RT [3]. Notably, RT is not a direct trigger for HSV reactivation, however, the immunocompromised state and accompanying anti-cancer therapy may be linked to an increased infection risk in CLL patients [4]. Therefore, it is pertinent always to consider HSV-related conditions, mainly when aggressive lymphadenopathy occurs with lymphomas and leukemias [4]. We report an unusual case of newly discovered inguinal lymphadenopathy in an immunocompromised patient who initially presented with shortness of breath and was successfully managed with antiviral therapy.

## Case presentation

A 62-year-old female patient presented to our hospital with complaints of progressive shortness of breath on exertion for two weeks. During the same period, she reported dysuria and pelvic discomfort as well as fatigue and 18-pound weight loss over 2 months. Systemic review was only significant for subjective fever but the patient denied chest pain, cough, hemoptysis, headache, abdominal pain, nausea, vomiting, or diarrhea. She had a medical history of CLL/SLL on acalabrutinib, obesity, and hyperlipidemia, while surgical history was only relevant for left chest wall Mediport placement and cholecystectomy. She was previously on antiviral prophylaxis while she was on acalabrutinib, however, she was not compliant with her medication. She reported no history of smoking, drinking, or recreational drug use. She also denied any recent travel or animal exposure.

On physical examination, the patient had a temperature of 102.5F, blood pressure of 107/65 mmHg, heart rate of 105 beats per minute, and respiratory rate of 20 breaths per minute. The abdomen was soft, non-tender, nondistended, and without organomegaly. Bilateral inguinal lymph nodes were noted to be enlarged (up to 4 cm in size), hard, fixed, and tender to palpation. The rest of the examination was unremarkable. Her initial laboratory results were significant for elevated white blood cell (WBC) count of 24860 /mm^3^ with 82.3% neutrophils, hemoglobin 8.9 g/dL, platelet count of 419000 /mm^3^, blood urea nitrogen (BUN) 6 g/dL, creatinine 0.56 g/dL, alkaline phosphatase (ALP) 144 U/L, albumin level 2.8 g/dL total bilirubin 0.5 mg/dL (see Table 1 for reference range). At the same time, urinalysis was deranged with proteinuria of 100 mg/dL, and pyuria with WBC count 16 /HPF, obtained from a good sample with squamous cells <5/HPF.

**Table 1:** Initial laboratory values on hospital admission

Laboratory Values	Measured Value	Reference Range
Hematologic values		
Leucocyte count	24860 /mm^3^	4000-11000 /mm^3^
Neutrophils	82.3%	54%-62%
Hemoglobin	8.9 g/dL	12.0-16.0 g/dL
Platelet count	419000 /mm^3^	150000-400000 /mm^3^
Serum values		
Sodium	134 mEq/L	135-145 mEq/L
Potassium	3.4 mEq/L	3.5-5.4 mEq/L
Blood urea nitrogen	6 mg/dL	8-20 mg/dL
Creatinine	0.56 mg/dL	0.70-1.20 mg/dL
Alkaline phosphatase	144 U/L	20-130 U/L
Aspartate aminotransferase	27 U/L	0-35 U/L
Alanine aminotransferase	18 U/L	0-40 U/L
Total bilirubin	0.5 mg/dL	0.0-1.5 mg/dL
Albumin level	2.8 g/dL	3.5-5.0 g/dL
Urinalysis (UA)		
UA protein	100 mg/dL	Negative
UA white cell count	16 /HPF	0-5 /HPF
UA red cell count	6 /HPF	0-2 /HPF
UA nitrites	Negative	Negative
UA leucocyte esterase	Trace	Negative

**Figure 1:**
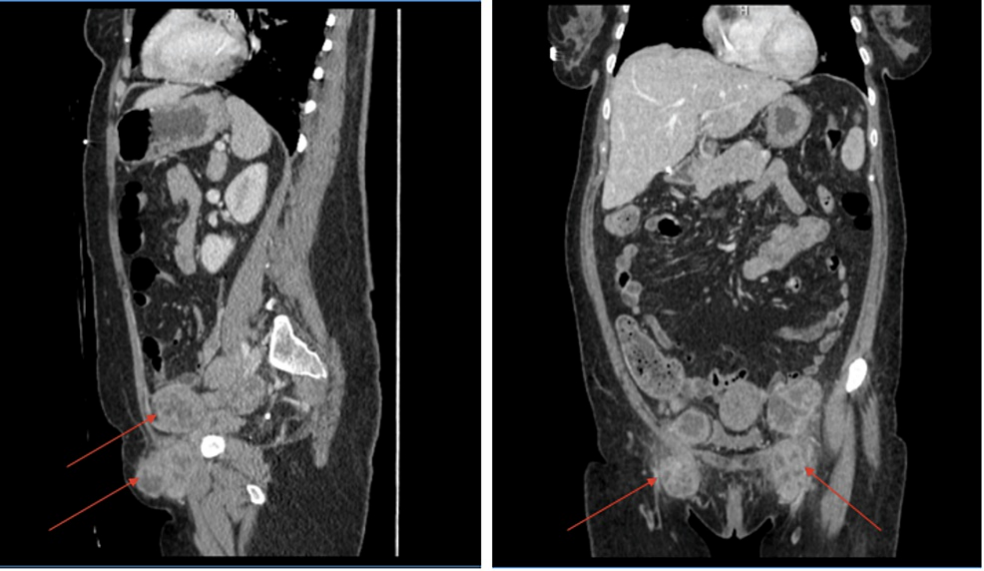
Bilateral iliac chain and inguinal lymphadenopathy with central hypodensities likely representing areas of necrosis (red arrows)

Computed tomography (CT) scan of the abdomen and pelvis with intravenous contrast demonstrated left liver lobe hypodensities, new bulky bilateral iliac chains, and inguinal lymphadenopathy with central hypodensities likely representing areas of necrosis (see Figure 1). Blood and urine cultures were sent, and the patient was empirically started on cefepime 1g every six hours. The blood and urine cultures were negative at 48 hours so cefepime was discontinued.

**Figure 2:**
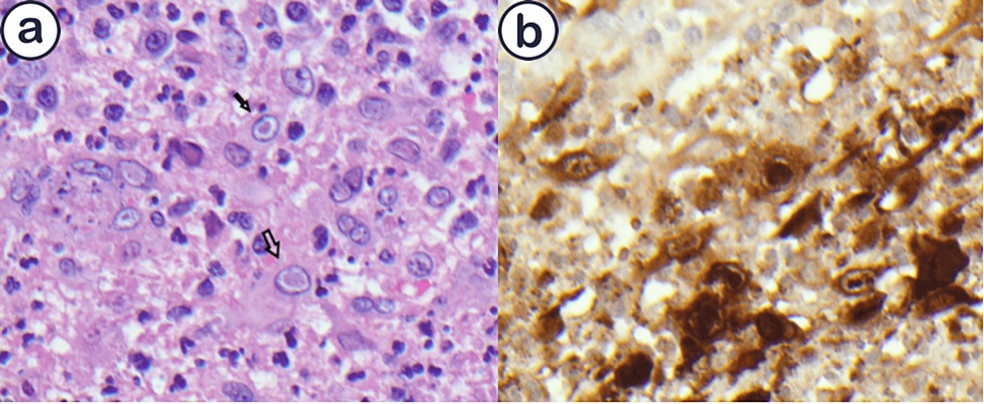
(a) Multinucleate cells with ground glass nuclei and Cowdry type A intranuclear inclusions; (b) Immunohistochemical stain showing HSV organisms

On the second day of admission, an excisional biopsy of bilateral deep inguinal nodes was performed. The histopathology revealed multinucleate lymphocytic cells with ground glass nuclei and Cowdry type A intranuclear inclusions surrounding microabscesses (Figures 2a-2b).

Immunohistochemical stains for HSV-1 and -2 were positive, consistent with active HSV infection and there was no RT nor CLL progression. In addition, flow cytometry was performed, and there was no evidence of diffuse large B-cell lymphoma (DLBCL). Serology was positive for HSV-1 and -2 IgG, while IgM antibodies were negative. Lymph node gram stain, aerobic and anaerobic, acid-fast bacilli (AFB), and fungal cultures all yielded no growth of organisms.

The patient clinically improved with the resolution of fever, tachycardia, and leukocytosis and was eventually discharged on valacyclovir three times daily for four weeks for HSV necrotizing lymphadenopathy. A follow-up outpatient visit one month later showed a marked reduction in the size of the affected lymph nodes and improved local pain and swelling. She was maintained on long-term suppression with valacyclovir 500mg twice daily after completion of her induction course; however, she stopped taking the medication after a few months. Her immunoglobulin levels were abnormally low so she was maintained on monthly IV Immunoglobulin (0.4g/kg) infusions to prevent subsequent infections. The dosing interval was also altered based on her immunoglobulin levels.

## Discussion

HSVs are giant enveloped DNA viruses from the alpha group of the Herpesviridae family. Primarily, they cause characteristic vesicular and pustular skin lesions that tend to rupture, forming ulcers and crusts [5]. In addition, they can establish latency in the sensory neurons, which can later reactivate and cause repeated infections [5]. Skin manifestations may be absent in immunocompromised patients with CLL/SLL, and affected patients may show non-specific symptoms such as fever, night sweats, and lymphadenopathy, which may mimic classic B-symptoms that are commonly associated with both Hodgkin and non-Hodgkin lymphomas [6].

Retrospectively, when looking at our patient's case, she had no oral or genital ulcers and no other skin lesions. She had been on a steady regimen of acalabrutinib chemotherapy for her CLL/SLL for almost a year with no significant changes made to her chemotherapy. She was not consistent with the use of acyclovir prophylaxis for HSV or VZV. Notably, the National Comprehensive Cancer Network (NCCN) provides considerations for *Pneumocystis jiroveci* pneumonia (PJP) and other viral prophylaxis for patients being treated with Bruton tyrosine kinase (BTK) inhibitors [7].

The pathogenesis of HSV infection in CLL/SLL patients remains unclear. Studies have however shown that elevated levels of herpes virus entry protein A (Hve-A) are produced in CLL patients [4], which contribute to viral entry via direct binding to HSV glycoprotein D molecule [8]. Furthermore, the increased risk of infection could also be attributable to CLL B cells functioning as HSV antigen-presenting cells [9], an ineffective natural killer response, and a T cell-mediated cytotoxic response [9].

A review of literature published in 2016 described four cases of CLL/SLL with superimposed HSV infection that mimicked the development of aggressive lymphoma via Richter's transformation [10]. This phenomenon occurs in 5-10% of patients and is characterized by the blastic transformation of CLL/SLL into DLBCL [11]. Although DLBCL is the most common outcome, RT may also describe the development of Hodgkin lymphoma (HL), plasmablastic lymphoma, or B-lymphoblastic leukemia/lymphoma [11]. Other CLL/SLL complications are briefly summarized in Table 2 below. On the other hand, HSV may affect the lymph nodes and can demonstrate clinical and pathological features similar to this transformation. There was an excellent response to antiviral therapy, with three out of four patients alive at the time of follow-up at 10 months [10]. 

**Table 2:** CLL/SLL complications CLL/SLL: chronic lymphocytic leukemia and small lymphocytic lymphoma

CLL/SLL Complications	Comments
Infections	Bacterial - Staphylococcus aureus, Streptococcus pneumoniae, Haemophilus influenzae, Escherichia coli, Klebsiella pneumoniae, and Pseudomonas aeruginosa; viral - Listeria, Mycobacteria, Nocardia, Candida, Aspergillus, Cryptococcus, Pneumocystis, Herpes simplex virus, Varicella-zoster virus and Cytomegalovirus
Autoimmune cytopenias	Autoimmune hemolytic anemia, immune-mediated thrombocytopenia, pure red blood cell aplasia
Second cancers	Acute myeloid leukemia, myelodysplastic syndromes, melanoma, gastrointestinal cancer, breast cancer, lung cancer, non-melanoma skin cancer, prostate cancer, kidney cancer, bladder cancer, head and neck cancers.
Richter transformation (<10% cases)	Diffuse large-B cell lymphoma (95%), Hodgkin lymphoma (5%)
Tumor lysis syndrome	Destruction of large tumor cell burden by chemotherapy leads to the release of cell byproducts and results in hyperphosphatemia, hypocalcemia, hyperkalemia, and renal insufficiency
Leukostasis	Severe leukocytosis (>400,000 /mm^3^) with features of decreased tissue perfusion

Acalabrutinib is an oral, second-generation selective BTK inhibitor indicated for the treatment of adult patients with CLL/SLL. It is also currently FDA-approved for the treatment of Waldenstrom macroglobulinemia [12]. Acalabrutinib can also be used as a less-aggressive alternative for treatment-naive mantle cell lymphoma (MCL) or in combination with rituximab as a first-line therapy for older patients with MCL in a phase II clinical trial [13,14]. Different trials evaluating both treatment-naive (phase III ELEVATE-TN; NCT02475681) and relapsed/refractory (phase III ASCEND; NCT02970318) CLL/SLL patients showed that acalabrutinib led to significant progression-free survival compared to standard of care; however, there is a lack of mature overall survival data [15,16]. Additionally, patients on this therapy are also at risk of infectious complications due to factors such as inhibition of IL-2-inducible T-cell kinase, macrophage impairment, and neutrophil dysfunction [17,18]. In the phase III ELEVATE-TN study, the most common adverse effects were diarrhea, headache, fatigue, arthralgia, cough, and upper respiratory tract infection, occurring mainly during the first year of therapy [12]. Lymphadenopathy or viral lymphadenitis was not described as an adverse event, however. 

In our case, the patient was treated with oral valacyclovir 1g twice daily for four weeks followed by long-term suppression with 500mg twice daily dosage. However, there are no established guidelines for treatment, as different institutions have different regimens. In a limited study of five patients with HSV lymphadenitis in the setting of CLL, outcomes were comparable between four patients who received no treatment and one who received antiviral therapy with no sequelae of worsening disease [19]. At present, there are no clear guidelines for HSV lymphadenitis treatment as regards criteria for patient selection as well as duration of treatment. Therefore, further work is needed to evaluate the role of antiviral therapy in managing HSV lymphadenitis.

Furthermore, the NCCN provides stratification for infection risk in cancer patients. Per NCCN guidelines, CLL is classified as an intermediate risk with recommendations to consider HSV/VZV prophylaxis [20]. This also depends on additional risk factors - whether patients are on myelosuppression treatment and their seropositivity/vaccination history. Furthermore, for patients who are on immunosuppressive therapies such as BTK inhibitors for CLL/SLL, NCCN recommends considering PJP and HSV/VZV prophylaxis for patients with additional risk factors while receiving therapy [20].

## Conclusions

Herpes simplex virus necrotic lymphadenitis occurs rarely in patients with CLL/SLL, and its diagnosis should be considered for patients with progressive lymph node enlargement and systemic symptoms that are concerning for infection. Our case emphasizes the importance of considering infectious etiologies as a possible cause of lymphadenopathy in immunocompromised patients, including those with CLL/SLL. Routine HSV prophylaxis with duration in these patients undergoing treatment with acalabrutinib is currently lacking and should be a topic for further research.
